# Comparing Patient Risk Factor-, Sequence Type-, and Resistance Locus Identification-Based Approaches for Predicting Antibiotic Resistance in Escherichia coli Bloodstream Infections

**DOI:** 10.1128/JCM.01780-18

**Published:** 2019-05-24

**Authors:** Derek R. MacFadden, Roberto G. Melano, Bryan Coburn, Nathalie Tijet, William P. Hanage, Nick Daneman

**Affiliations:** a University of Toronto, Division of Infectious Diseases, Toronto, Ontario, Canada; b Harvard T. H. Chan School of Public Health, Boston, Massachusetts, USA; c Public Health Ontario Laboratory, Toronto, Ontario, Canada; Medical College of Wisconsin

**Keywords:** antibiotic resistance, prediction, rapid diagnostics

## Abstract

Rapid diagnostic tests for antibiotic resistance that identify the presence or absence of antibiotic resistance genes/loci are increasingly being developed. However, these approaches usually neglect other sources of predictive information which could be identified over shorter time periods, including patient epidemiologic risk factors for antibiotic resistance and markers of lineage. Using a data set of 414 Escherichia coli isolates recovered from separate episodes of bacteremia at a single academic institution in Toronto, Ontario, Canada, between 2010 and 2015, we compared the potential predictive ability of three approaches (epidemiologic risk factor-, pathogen sequence type [ST]-, and resistance gene identification-based approaches) for classifying phenotypic resistance to three antibiotics representing classes of broad-spectrum antimicrobial therapy (ceftriaxone [a 3rd-generation cephalosporin], ciprofloxacin [a fluoroquinolone], and gentamicin [an aminoglycoside]). We used logistic regression models to generate model receiver operating characteristic (ROC) curves. Predictive discrimination was measured using apparent and corrected (bootstrapped) areas under the curves (AUCs). Epidemiologic risk factor-based models based on two simple risk factors (prior antibiotic exposure and recent prior susceptibility of Gram-negative bacteria) provided a modest predictive discrimination, with AUCs ranging from 0.65 to 0.74. Sequence type-based models demonstrated strong discrimination (AUCs, 0.83 to 0.94) across all three antibiotic classes. The addition of epidemiologic risk factors to sequence type significantly improved the ability to predict resistance for all antibiotics (*P* < 0.05). Resistance gene identification-based approaches provided the highest degree of discrimination (AUCs, 0.88 to 0.99), with no statistically significant benefit being achieved by adding the patient epidemiologic predictors. In summary, sequence type or other lineage-based approaches could produce an excellent discrimination of antibiotic resistance and may be improved by incorporating readily available patient epidemiologic predictors but are less discriminatory than identification of the presence of known resistance loci.

## Abstract (precis)

Rapid diagnostic tests for antibiotic resistance that identify the presence or absence of antibiotic resistance genes/loci are increasingly being developed. However, these approaches usually neglect other sources of predictive information which could be identified over shorter time periods, including patient epidemiologic risk factors for antibiotic resistance and markers of lineage.

## INTRODUCTION

Antibiotic resistance is a growing public health challenge globally and threatens to undo major advances in the treatment of bacterial infections (1, 2). Infections due to antibiotic-resistant organisms are associated with increased morbidity and mortality, and this may be due in part to inadequate initial empirical antibiotic therapy (3, 4). In severe infections, including septic shock and bacteremia, the time to the receipt of adequate antibiotic therapy has been shown to be a predictor of clinical outcomes, including mortality (5, 6). In order to improve these outcomes by ensuring therapeutic adequacy as early as possible, clinicians commonly prescribe antimicrobials with activity against a wider range of pathogens than is necessary for most infections, even when very targeted therapy would be sufficient.

Rapid microbial diagnostics, capable of early identification of infecting pathogens and their antibiotic resistance profiles, are increasingly being developed (7, 8). These tests offer the promise of reducing unnecessary antibiotic use, as well as improving the time to early adequate therapy. Unfortunately, many rapid diagnostic approaches are not uniformly capable of identifying species and/or a range of antibiotic resistance mechanisms and are frequently dependent upon cultured samples (9). Genome-based approaches that could obviate prolonged specimen culture, as well as provide detailed species, lineage, and antimicrobial resistance information about the infecting pathogens, are being developed. The use of whole-genome sequencing (WGS) to identify loci of resistance using large resistance databases has been the predominant approach (10, 11).

One limitation of existing sequencing platforms is the dependence on relatively time- and bioinformatics-intensive pipelines. New sequencing platforms, including the Oxford Nanopore platform, can provide rapid real-time sequencing, but even these techniques can take hours to accurately identify resistance loci/mutations (10, 12, 13). One option which combines both speed and genomic analysis is the lineage-calling method. This method is somewhat analogous to multilocus sequence typing (MLST), where a pathogen can be matched (using k-mer-based approaches) to a database of known lineages and susceptibility patterns in order to predict antibiotic resistance/susceptibility. This novel approach of identifying resistance-associated sequence elements (RASE) requires considerably less genomic processing/bioinformatics and can identify a probable phenotype within 1 to 5 min after the start of sequencing (14). Reducing the sequencing and bioinformatics time to such a short duration with minimal computational requirements means that affordable and portable sequence-based approaches could be employed to impact initial empirical therapy in the clinic or hospital. However, the success of this technique relies on the association between the lineage (e.g., the sequence type [ST] or phylogroup) and the antibiotic susceptibility profile, which remains to be demonstrated across scale for many pathogens, including common Gram-negative bacteria, such as Escherichia coli.

Genomic methods, either locus- or lineage-calling methods, are inherently imperfect. They focus on genetic information and do not provide direct measures of the phenotype (11). One approach to improve the predictive accuracy of both techniques is combining them with patient historical and epidemiologic predictors of resistance, which may also be associated with the antibiotic resistance phenotype. Use of a technique that combines important antibiotic resistance predictors from readily available electronic medical records and genomic data may improve the predictive ability and, thus, the clinical utility (15, 16).

In this paper, we sought to evaluate the ability of combinations of three approaches, (i) patient epidemiologic characteristic-based, (ii) pathogen sequence type-based, and (iii) resistance locus identification-based approaches, in order to predict antibiotic resistance in Escherichia coli bloodstream infections.

## MATERIALS AND METHODS

Bloodstream isolates that were recovered from 414 unique episodes of E. coli bacteremia between the years 2010 and 2015 at a single 1,325-bed tertiary care academic center in Toronto, Ontario, Canada (Sunnybrook Health Sciences Centre), and that had been prospectively stored were identified for whole-genome sequencing. The antibiotic susceptibilities of the infecting E. coli isolates were determined on the basis of clinical phenotypic susceptibility testing, which occurred at the time of initial culture/isolation. These were performed using a Vitek 2 system with Clinical Laboratory Standards Institute (CLSI) breakpoints. Since the introduction of the M100-S20 CLSI update in 2010, the breakpoints for ceftriaxone, gentamicin, and ciprofloxacin have not changed at this institution. We evaluated susceptibility to a 3rd-generation cephalosporin (ceftriaxone), a fluoroquinolone (ciprofloxacin), and an aminoglycoside (gentamicin). We chose these three antibiotics as representatives of their classes for the following reasons: (i) we have previously evaluated the utility of specific epidemiologic predictors for these antibiotics; (ii) they each represent one of the three major classes of broad-spectrum antimicrobials commonly used as backbones for therapy focused against Gram-negative bacteria; and (iii) in general, these specific antimicrobials are correlated with resistance to other antibiotics of the same class. The resistance outcomes were summarized as a binary variable with either a susceptible or a nonsusceptible state (nonsusceptible could include either the intermediate or resistant phenotype). For simplicity, we refer to nonsusceptible as “resistant” throughout the text.

### Genome sequencing and resistance locus identification.

Isolates were first grown in culture from glycerol stocks. Genomic DNA was extracted using a QIAamp DNA minikit (Qiagen) according to the manufacturer’s instructions. The DNA concentration was determined by use of a Qubit (version 2.0) fluorometer (Thermo Fisher Scientific), and DNA samples were stored at −20°C until further processing. The sequencing library was prepared using an Illumina Nextera XT DNA library preparation kit (Illumina) per the manufacturer’s instructions. An Agilent 2100 bioanalyzer was used to determine the quality and quantity of the DNA library. Whole-genome sequencing (WGS) was performed by Illumina paired-end sequencing using a NextSeq500 desktop sequencer (version 2). An average of 100× coverage was achieved. *De novo* assemblies of Illumina reads were prepared using the Spades (version 3.5) program.

Resistance genes were detected using the raw reads to query the ResFinder database using the SRST2 program with default settings (17). Genetic loci of resistance were included as binary values, with the presence of a gene/mutation possibly conferring resistance (intermediate or resistant phenotypes) to the antibiotic of interest being denoted 1 and the absence/unknown presence of such a gene/mutation being denoted 0. Fluoroquinolone resistance was combined into a single variable reflecting a mutation in both *gyrA* and *parC*. All the loci found in our WGS analysis related to resistance to the studied antimicrobials were included: *aac(3)*, *aac(6ʹ)*, and *aadB* for gentamicin; for ciprofloxacin, a single mutation or double mutations in the *gyrA* quinolone resistance-determining region (QRDR; positions S83 and D87), a single mutation or double mutation in the *parC* QRDR (positions S80 and E84), and the presence of plasmid-mediated *qnrS1*, *oqxAB*, or *qepA* were initially considered (although these plasmid-mediated mechanisms were ultimately not considered in the model, given their virtual absence from these evaluated isolates). Genes for broad-spectrum (e.g., *bla*_TEM-1_, *bla*_SHV-1_, or *bla*_OXA-1_), extended-spectrum (*bla*_CTX-M_ alleles), and plasmid-mediated AmpC (*bla*_CMY_ alleles) β-lactamases were included in the analysis for ceftriaxone resistance.

We used genetic features to assign each isolate to a particular multilocus sequence type (MLST). The MLST schemes were carried out *in silico* at the Centre for Genomic Epidemiology website using assembled sequences (18). We used sequence type as a categorical variable, including all STs corresponding to an approximately 1% prevalence or greater (4 or more isolates with the particular sequence type).

A maximum-parsimony tree based on 7 concatenated genes (used for MLST assignment) was generated using BioNumerics (version 6.6) software, and the most common STs are labeled. A single isolate with a divergent sequence type (ST5969) was excluded from Fig. S1 in the supplemental material, given its long branch length.

### Epidemiologic predictors.

In alignment with previous work, we identified and included in our epidemiologic factor-based models two strong predictors of antibiotic resistance: (i) the resistance of the most recently isolated Gram-negative bacterium (from any site) to the antibiotic of interest (up to 1 year from the time of collection of the sample for the index bacterial culture) and (ii) exposure to the same class of antibiotics within the previous 90 days (from the time of collection of the sample for the index culture) (15). For the 3rd-generation cephalosporin, prior exposures to both penicillins and cephalosporins were considered binary indicators. For ciprofloxacin (a fluoroquinolone) and gentamicin (an aminoglycoside), exposure to any agent in the respective class was considered a binary exposure variable. We identified these two epidemiologic predictors of antibiotic resistance from admissions corresponding to the bacterial isolates. We chose to consider prior antimicrobial exposure on a class level as a relevant predictor of resistance for the following reasons: (i) consideration of all individual antimicrobials for a given class to be independent predictors would require too many predictors for model fit; (ii) we generally believe that selective pressure acts as a class-dependent effect (although coselection can occur); and (iii) as noted above, we have previously evaluated class exposure as a predictor variable, and it has a strong predictive power. Data were collected from a comprehensive and secure prospective clinical database as previously described (15). There were less than 0.3% missing values across all predictors.

### Prediction models.

We specified prediction models *a priori* to correspond to (i) epidemiologic predictors alone (prior isolate and prior antibiotic use), (ii) sequence type alone, (iii) sequence type with the addition of epidemiologic predictors, (iv) resistance loci alone, or (v) resistance loci with the addition of epidemiologic predictors. Logistic regression models were constructed with the relevant epidemiologic and sequence-based predictors (sequence type or resistance loci). In order to evaluate model discrimination, receiver operating characteristic (ROC) curves were generated and apparent area under the curve (AUC) values, along with bootstrapped optimism-adjusted AUCs (1,000 bootstraps), were calculated. The DeLong approach for paired ROC curves was used to compare apparent model AUCs with or without epidemiologic predictors, to evaluate whether they contributed significantly to the model fit (19). Model calibration and goodness of fit were evaluated using the Hosmer-Lemeshow test (*P* values are provided in Table S1). All analyses were performed using R (version 1.1.383). This study was conducted in accordance with principles from the TRIPOD statement (20).

Research ethics board approval for this study was obtained at Sunnybrook Health Sciences Center.

### Accession number(s).

All genomes were submitted to GenBank (BioProject PRJNA521038).

## RESULTS

Across the patients with 414 unique episodes of bacteremia, the mean age was 65.6 years, with 51% of the episodes occurring in women and the majority occurring on nonsurgical services (Table 1). Phenotypic resistance to the 3rd-generation cephalosporin (ceftriaxone), fluoroquinolone (ciprofloxacin), and aminoglycoside (gentamicin) was present in 12%, 27%, and 13% of the isolates, respectively (Table 1). Exposure to the antibiotic class of interest in the 90 days prior to an episode of bacteremia was common in resistant isolates, occurring in over 65% of cases for all evaluated antibiotics. Prior cultures with organisms resistant to the same class in the previous year were less common, occurring in at least 22% of episodes (with resistant isolates) for all antibiotic classes evaluated. The most common sequence type was ST131 (21%) and was more prevalent in resistant isolates than in susceptible isolates for all three antibiotics (41% to 54%). The most common beta-lactam resistance mechanism identified was *bla*_TEM-1_, which encodes a broad-spectrum β-lactamase, present in 41% of all isolates. The most common extended-spectrum β-lactamase gene family identified was *bla*_CTX-M_, present in 10% of all isolates. Fluoroquinolone resistance due to point mutations in *gyrA* (positions S83 and D87) and *parC* (S80) was found in 37% and 28% of all isolates, respectively, and was modeled in combination (requiring at least one mutation in both *gyrA* and *parC*). Only 3 types of aminoglycoside-modifying enzymes were detected, and all of them were able to inactivate gentamicin, with *aac(3)* being the most prevalent (12%) (Table 1).

**TABLE 1:** Epidemiologic characteristics, sequence types, and genetic mechanisms of resistance for the entire cohort as well as by resistance to a particular antibiotic representative of a corresponding class^*a*^

Characteristic	Value for the following isolates:			
	All isolates (*n* = 414)	Ceftriaxone resistant (*n* = 51)	Ciprofloxacin resistant (*n* = 113)	Gentamicin resistant (*n* = 54)
% of all isolates		12	27	13
Mean age (yr)	65.6	66.8	69.8	67.7
No. (%) of isolates from female patients	213 (51)	23 (45)	52 (46)	18 (33)
No. (%) of isolates from patients on a nonsurgical service	328 (79)	36 (71)	94 (83)	44 (81)
No. (%) of isolates from patients with:				
Prior antibiotic exposure (same class) in 90 days		37 (73)	75 (66)	35 (65)
Prior resistant culture (same class) in last yr		12 (24)	31 (27)	12 (22)
No. (%) of isolates of the following MLST sequence type:				
ST131	87 (21)	21 (41)	61 (54)	28 (52)
ST95	58 (14)	2 (4)	2 (2)	2 (4)
ST73	57 (14)	1 (2)	0	0
ST69	22 (5)	0	0	3 (6)
ST127	15 (4)	0	0	1 (2)
ST1193	14 (3)	0	14 (12)	2 (4)
ST405	9 (2)	6 (12)	9 (8)	6 (11)
Other	152 (37)	21 (41)	27 (24)	12 (22)
No. (%) of isolates with the indicated mechanism of resistance to the following antibiotics:				
Beta-lactams				
*bla*_CMY_	12 (3)	10 (20)	6 (5)	5 (9)
*bla*_CTX-M_	42 (10)	39 (76)	37 (33)	13 (24)
*bla*_SHV_	7 (2)	1 (2)	1 (1)	1 (2)
*bla*_OXA_	24 (6)	17 (33)	19 (17)	10 (19)
*bla*_TEM_	169 (41)	18 (35)	72 (64)	42 (78)
Fluoroquinolones:				
*gyrA*	155 (37)	41 (80)	111 (98)	47 (87)
*parC*	116 (28)	38 (75)	110 (97)	36 (67)
Aminoglycosides				
*aac(3)*	49 (12)	12 (24)	34 (30)	48 (89)
*aac(6′)*	21 (5)	16 (31)	18 (16)	10 (19)
*aadB*	4 (1)	4 (8)	3 (3)	3 (6)

a The antibiotic classes represented were 3rd-generation cephalosporins (ceftriaxone), fluoroquinolones (ciprofloxacin), and aminoglycosides (gentamicin).

**FIG 1:**
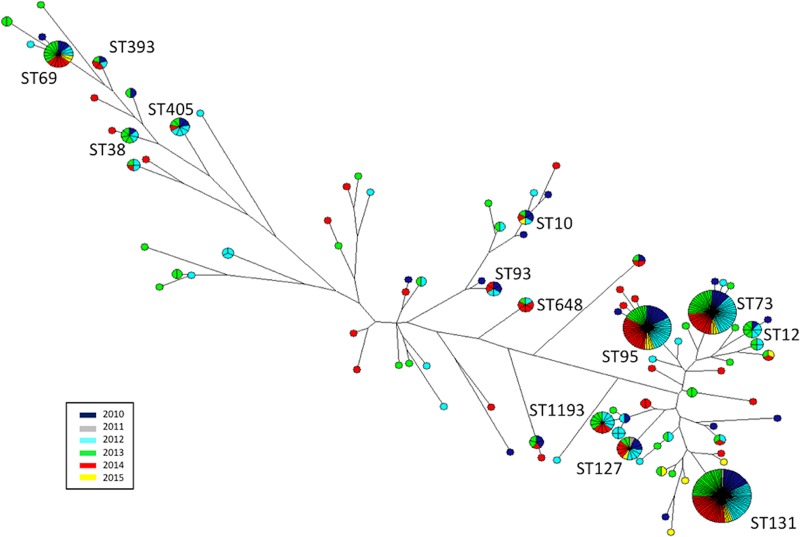
Maximum-parsimony tree with the corresponding sequence types (labeled) and years of isolate recovery (colors) for all E. coli isolates.

A maximum-parsimony tree was assembled (Fig. 1) and demonstrated the predominance of a few major sequence types with related housekeeping genes. The most common was ST131, accounting for 21% of the sample.

**FIG 2:**
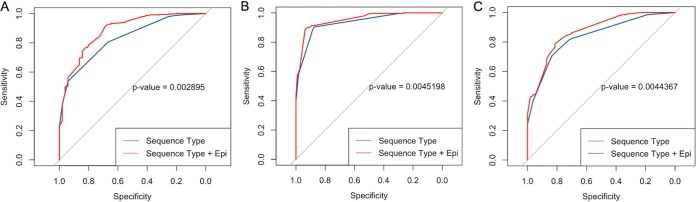
Receiver operating characteristic curves for the sequence type-based approach with or without epidemiologic predictors (Epi) for ceftriaxone (a 3rd-generation cephalosporin) (A), ciprofloxacin (a fluoroquinolone) (B), and gentamicin (an aminoglycoside) (C).

For 3rd-generation cephalosporin resistance, a baseline epidemiologic predictor only-based model (prior antibiotic use and prior resistance) yielded an apparent area under the curve (AUC) of 0.74 (95% confidence interval [CI], 0.66 to 0.82), whereas a sequence type only-based model yielded an AUC of 0.83 (95% CI, 0.78 to 0.88) (Table 2). The addition of epidemiologic predictors to the sequence type-based model (Fig. 2;Table 2) increased the AUC to 0.88 (95% CI, 0.83 to 0.93), and this was statistically significant (*P* = 0.003). A β-lactamase locus only-based model generated AUCs of 0.88 to 0.98, with the *bla*_CTX-M_ and *bla*_CMY_ predictors providing the greatest increases in AUC. The addition of epidemiologic predictors to a full β-lactamase locus-based model yielded an AUC of 0.98 (95% CI, 0.96 to 1), which was not a statistically significant change from that for a full β-lactamase locus-based model alone (Table 2; see also Fig. S1 in the supplemental material).

**TABLE 2:** Antibiotic resistance prediction performance, as measured by the AUC, for gene- and sequence typing-based approaches with or without the addition of epidemiologic risk factors for resistance

Antibiotic model (*n* = 414)	With epidemiologic predictors			Without epidemiologic predictors		
	Apparent AUC	AUC 95% CI	Corrected AUC	Apparent AUC	AUC 95% CI	Corrected AUC
Ceftriaxone (396 isolates tested)						
Baseline (Epi^*a*^ predictors alone)	0.74	0.66–0.82	0.72			
ST^*b*^^,^^*c*^	0.88	0.83–0.93	0.85	0.83	0.78–0.88	0.81
* *β-Lactamases						
* bla*_CTX-M_	0.93	0.87–0.98	0.91	0.88	0.82–0.94	0.88
* bla*_CTX-M_ + *bla*_CMY_	0.98	0.95–1	0.97	0.97	0.94–1	0.97
* bla*_CTX-M_ + *bla*_CMY_ + *bla*_OXA_	0.97	0.94–1	0.96	0.97	0.94–1	0.97
* bla*_CTX-M_ + *bla*_CMY_ + *bla*_OXA_ + *bla*_TEM_	0.98	0.96–1	0.97	0.98	0.96–1	0.97
* bla*_CTX-M_ + *bla*_CMY_ + *bla*_OXA_ + *bla*_TEM_ + *bla*_SHV_	0.98	0.96–1	0.97	0.97	0.93–1	0.96
Ciprofloxacin (414 isolates tested)						
Baseline (Epi predictors alone)	0.68	0.63–0.73	0.67			
ST^*b*^^,^^*c*^	0.95	0.94–0.97	0.94	0.94	0.91–0.96	0.93
* gyrA + parC* mutations	0.99	0.98–1	0.99	0.98	0.97–1	0.98
Gentamicin (414 isolates tested)						
Baseline (Epi predictors alone)	0.65	0.58–0.72	0.64			
ST^*b*^^,^^*c*^	0.87	0.82–0.92	0.83	0.84	0.79–0.89	0.8
* *Aminoglycoside acyltransferases						
* aac(*3*)*	0.96	0.93–1	0.96	0.94	0.90–0.99	0.94
* aac(*3*) + aac(6′)*	0.96	0.93–1	0.96	0.94	0.90–0.99	0.94
* aac(*3*) + aac(6′) + aadB*	0.98	0.95–1	0.97	0.97	0.94–1	0.97

a Epi, epidemiologic.

b ST, sequence type (multilocus).

c Significant difference between AUCs with epidemiologic predictors and AUCs without epidemiologic predictors (*P* < 0.05).

For fluoroquinolone resistance, a baseline epidemiologic predictor-based model yielded an apparent AUC of 0.68 (95% CI, 0.63 to 0.73) (Table 2). A sequence type only-based model yielded an AUC of 0.94 (95% CI, 0.91 to 0.96), and the addition of epidemiologic predictors (Fig. 2) to this model increased the AUC to 0.95 (95% CI, 0.94 to 0.97), which was a statistically significant increase (*P* = 0.005) (Fig. 2; Table 2). A model based on the presence of the *gyrA* and *parC* mutations had an AUC of 0.98 (95% CI, 0.97 to 1), which was not significantly changed by the addition of epidemiologic predictors (Table 2; Fig. S1).

For aminoglycoside (gentamicin) resistance, a baseline epidemiologic predictor-based model yielded an apparent AUC of 0.65 (95% CI, 0.58 to 0.72) (Table 2). A sequence type only-based model yielded an AUC of 0.84 (95% CI, 0.79 to 0.89), and the addition of epidemiologic predictors to this model (Fig. 2) increased the AUC to 0.87 (95% CI, 0.82 to 0.92), which was a statistically significant increase (*P* = 0.004) (Fig. 2; Table 2). A model based on the presence of aminoglycoside acyltransferase enzymes had AUCs ranging from 0.94 to 0.97 with the sequential addition of resistance loci (Table 2). Addition of epidemiologic predictors supported a minimal increase in AUCs ranging from 0.96 to 0.98, and these increases were not statistically significant (Fig. S1).

AUCs adjusted for optimism were all generally the same or modestly lower than the apparent AUCs for all models (Table 2).

## DISCUSSION

In this study, we evaluated three approaches to predicting antibiotic resistance in E. coli bloodstream infections: (i) an epidemiologic predictor-based approach, (ii) a pathogen sequence type-based approach, and (iii) a genetic resistance locus-based approach. We found that the sequence type-based approaches provided an excellent ability to predict antibiotic resistance in E. coli. Moreover, predictive discrimination could be improved using simple epidemiologic factors from electronic medical records. Lastly, we found that genetic resistance loci offered the greatest predictive discrimination and, in some instances, indicated susceptibility almost perfectly matching the phenotypic susceptibility.

Antibiotic resistance prediction is important, as it can both potentially improve the time to adequate therapy and reduce unnecessarily broad antimicrobial use and is therefore a tool for antibiotic stewardship (21). While a prediction is inherently imperfect compared to the results of phenotypic methods, an imperfect prediction may be able to improve on existing heuristic-based decision making for empirical antibiotic use (9). Our findings add to existing data on the use of genetic loci for predicting the antibiotic susceptibilities of clinical isolates of Gram-negative bacteria (11, 22). Specifically, they reinforce the suggestion that a nearly perfect prediction of phenotypic susceptibility is achievable for some antibiotics/classes and organisms. However, this approach requires that the locus responsible be known in advance, map one-to-one onto the resistance phenotype, and be readily detectable and distinguishable from related loci that do not confer resistance. Existing rapid whole-genome sequencing techniques, namely, the Oxford Nanopore platform, can generate real-time reads, but they suffer from relatively high error rates (10). This is nonideal for identifying resistance loci, particularly point mutations, such as those conferring fluoroquinolone resistance. However, approaches that can identify the lineage using Nanopore reads and k-mer-based analysis can be performed within minutes (14). This has recently been demonstrated for Streptococcus pneumoniae, where an existing k-mer database of strains linked with the resistance phenotype was used to rapidly match and predict the resistance phenotype within 1 to 5 min (14). Sequence typing represents a simple form of lineage calling, where related isolates can be grouped into particular strain types given the similarity of their housekeeping genes (23). There are few data on the use of lineage-calling approaches for predicting resistance in Gram-negative organisms, and it is unclear whether this approach would work for resistance mechanisms mediated by point mutations (24). This is because strain-independent selection effects may be the factor driving the proliferation of fluoroquinolone-resistant clones. However, we found for all antibiotics evaluated that resistance could be accurately predicted using a sequence type, and this included resistance to those classes in which resistance is typically mediated through mutations (fluoroquinolones). This is likely related to the predominance of particular clonotypes (e.g., ST131) that contain point mutations conferring resistance to particular agents, including fluoroquinolones.

While use of the sequence type provided good discrimination, this approach was enhanced by the use of simple epidemiologic predictors which are immediately available at the time of initial empirical therapy through electronic medical record systems. This means that a combination of lineage type and epidemiologic information could theoretically be utilized in minutes to provide highly accurate predictions of resistance. The rapidity of lineage-calling approaches is, however, dependent upon the development of a reference database, as well as the ability to perform these analyses culture free and with a minimal DNA extraction/library preparation time. The latter issues of sample adequacy and preparation now represent the rate-limiting step in the implementation of rapid lineage-calling approaches. However, we have seen progressive reductions in the amount of time required for both DNA extraction and library preparation, and if these reductions continue as expected, then clinical use of the approach described here will be feasible. While our results apply directly to E. coli, it is likely that these findings would hold with other common Gram-negative organisms, with some possible antibiotic class-specific exceptions being those organisms able to rapidly modify porin/efflux pump expression, most notably, Pseudomonas aeruginosa (25), as well as AmpC-producing Enterobacteriaceae. It is worth noting that during our study period, the CLSI breakpoints for cephalosporins changed substantially (2010), and this may reduce the discriminatory ability of the models for ceftriaxone. However, because this occurred early on in the study period, it is unlikely to have a major impact, and in fact, our results should represent a lower limit of prediction for 3rd-generation cephalosporins.

It is also worth noting that this study was performed in a single tertiary care institution. This particular approach takes advantage of the fact that the diversity of E. coli lineages within institutions is limited compared with that in larger health network or geographic structures, with the result being a greater correlation of antibiotic resistance genes and lineage. Despite this possible challenge to generalizability, which itself needs further evaluation, it is reasonable to take advantage of these regional lineages and develop region-specific predictive approaches. These models could also account for temporal changes in local antibiotic resistance ecology.

In summary, the sequence type with or without relevant epidemiologic predictors can provide excellent discrimination of antibiotic resistance in E. coli isolates causing bloodstream infections in a single institution. The use of these two approaches combined offers the promise of predicting antibiotic resistance in the context of initial antibiotic therapy selection.

## Supplementary Material

Supplemental file 1
