# Supplementary material for: Comparing Patient Risk Factor-, Sequence Type-, and Resistance Locus Identification-Based Approaches for Predicting Antibiotic Resistance in Escherichia coli Bloodstream Infections
Source: J Clin Microbiol. 2019 May 24;57(6):e01780-18. doi: 10.1128/JCM.01780-18 (PMC6535602; doi:10.1128/JCM.01780-18)
Supplement: Supplemental file 1 [file JCM.01780-18-s0001.pdf]

Supplementary Table 1: Hosmer-Lemeshow p-values for evaluated models.

	Hosmer-Lemeshow P-value	
**Antibiotic Model (N=414)**	**Model Without Epi Predictors**	**Model With Epi Predictors**
**CEFTRIAXONE (Tested=396)**		
Baseline (Epi predictors Alone)	-	0.98
ST**	1	0.72
Beta-lactamases		
bla_{CTX}	1	0.99
bla_{CTX}+bla_{CMY}	1	0.99
bla_{CTX}+bla_{CMY}+bla_{OXA}	1	NA
bla_{CTX}+bla_{CMY}+bla_{OXA}+bla_{TEM}	1	0.78
bla_{CTX}+bla_{CMY}+bla_{OXA}+bla_{TEM}+bla_{SHV}	1	0.82
**CIPROFLOXACIN (Tested=414)**		
Baseline (Epi predictors alone)	-	1
ST**	1	0.93
gyrA + parC Mutations	1	1
**GENTAMICIN (Tested=414)**		
Baseline (Epi predictors Alone)	-	1
ST**	1	0.93
Aminoglycoside Acyl-transferases		
aac(3)	1	1
aac(3) + aac(6)	1	1
aac(3) + aac(6) + aadB	1	1

Interpret HL p-value with caution given limited number of binary/categorical predictors in some models.

Supplementary Table 2: Genetic resistance loci identified by isolate and multilocus sequence type (MLST).

MLST	CFTX	GENT	CIPRO	blaCMY	blaCTX	blaSHV	blaOXA	blaTEM	aac(3)	aac(6')	aadB	gyrA	parC
73	-	S	S	0	0	0	0	0	0	0	0	0	0
405	-	I+R	I+R	0	1	0	1	1	1	1	0	1	1
12	S	S	S	0	0	0	0	0	0	0	0	0	0
12	S	S	S	0	0	0	0	0	0	0	0	0	0
4238	S	S	S	0	0	0	0	0	0	0	0	0	0
95	S	I+R	S	0	0	0	0	1	1	0	0	1	0
404	S	S	S	0	0	0	0	1	0	0	0	1	0
4554	S	S	S	0	0	0	0	1	0	0	0	0	0
131	S	S	I+R	0	0	0	0	1	0	0	0	1	1
95	S	S	I+R	0	0	0	0	1	0	0	0	1	1
1431	S	S	I+R	0	0	0	0	1	0	0	0	1	1
131	I+R	S	I+R	0	1	0	0	0	0	0	0	1	1
131	-	S	I+R	0	0	0	0	1	0	0	0	1	1
131	I+R	I+R	I+R	0	1	0	1	0	1	1	0	1	1
NEW	S	S	S	0	0	0	0	0	0	0	0	0	1
73	S	S	S	0	0	0	0	0	0	0	0	0	0
1193	S	S	I+R	0	0	0	0	1	0	0	0	1	1
409	S	S	S	0	0	0	0	0	0	0	0	0	0
131	S	S	I+R	0	0	0	0	1	0	0	0	1	1
73	S	S	S	0	0	0	0	0	0	0	0	0	0
141	S	S	S	0	0	0	0	1	0	0	0	1	0
1284	I+R	I+R	I+R	1	1	0	1	0	1	1	0	1	1
95	S	S	S	0	0	0	0	0	0	0	0	0	0
52	-	S	S	0	0	0	0	0	0	0	0	1	0
73	S	S	S	0	0	0	0	0	0	0	0	0	0
131	S	S	S	0	0	0	0	1	0	0	0	1	0
95	S	S	S	0	0	0	0	1	0	0	0	0	0
38	S	S	S	0	0	0	0	1	0	0	0	0	0
73	S	S	S	0	0	0	0	0	0	0	0	0	0
131	S	I+R	I+R	0	0	0	0	1	1	0	0	1	1
393	S	S	I+R	0	0	0	0	0	0	0	0	1	1
73	S	S	S	0	0	0	0	0	0	0	0	0	0

28	S	S	S	0	0	0	0	0	0	0	0	0	0
135	S	S	S	0	0	0	0	0	0	0	0	0	0
131	-	S	I+R	0	0	0	0	1	0	0	0	1	1
73	S	S	S	0	0	0	0	0	0	0	0	0	0
14	S	S	S	0	0	0	0	0	0	0	0	0	0
95	S	S	S	0	0	0	0	0	0	0	0	0	0
73	S	S	S	0	0	0	0	0	0	0	0	0	0
73	S	S	S	0	0	0	0	0	0	0	0	0	0
1086	S	S	S	0	0	0	0	0	0	0	0	0	0
131	S	S	I+R	0	0	0	0	1	0	0	0	1	1
73	S	S	S	0	0	0	0	0	0	0	0	0	0
73	S	S	S	0	0	0	0	0	0	0	0	0	0
1193	S	S	I+R	0	0	0	0	0	0	0	0	1	1
131	-	S	I+R	0	0	0	0	1	0	0	0	1	1
404	S	S	S	0	0	0	0	1	0	0	0	0	0
73	S	S	S	0	0	0	0	0	0	0	0	0	0
1421	S	S	S	0	0	0	0	0	0	0	0	0	0
127	S	S	S	0	0	0	0	0	0	0	0	0	0
131	S	S	I+R	0	0	0	0	1	0	0	0	1	1
127	S	S	S	0	0	0	0	0	0	0	0	0	0
73	S	S	S	0	0	0	0	0	0	0	0	0	0
NEW	S	S	S	0	0	0	0	1	0	0	0	0	0
95	S	S	S	0	0	0	0	0	0	0	0	0	0
349	S	S	S	0	0	0	0	0	0	0	0	0	0
131	-	I+R	S	0	0	0	0	1	1	0	0	0	0
404	S	S	S	0	0	0	0	1	0	0	0	0	0
69	S	S	S	0	0	0	0	1	0	0	0	0	0
73	S	S	S	0	0	0	0	0	0	0	0	0	0
93	S	S	S	0	0	0	0	1	0	0	0	0	0
131	I+R	S	I+R	0	1	0	0	0	0	0	0	1	1
131	I+R	S	I+R	0	1	0	0	0	0	0	0	1	1
131	S	I+R	I+R	0	0	0	0	1	1	0	0	1	1
1284	I+R	S	I+R	0	1	0	1	0	0	1	0	1	1

95	S	S	S	0	0	0	0	1	0	0	0	0	0
95	S	S	S	0	0	0	0	1	0	0	0	0	0
95	-	S	S	0	0	0	0	1	0	0	0	0	0
144	S	S	S	0	0	0	0	1	0	0	0	0	0
363	S	S	S	0	0	0	0	0	0	0	0	0	0
73	S	S	S	0	0	0	0	0	0	0	0	1	0
58	S	S	S	0	0	0	0	0	0	0	0	1	0
73	S	S	S	0	0	0	0	1	0	0	0	0	0
NEW	S	S	S	0	0	0	0	0	0	0	0	0	0
95	S	S	S	0	0	0	0	0	0	0	0	0	0
1193	S	S	I+R	0	0	0	0	1	0	0	0	1	1
131	S	I+R	I+R	0	0	0	0	1	1	0	0	1	1
95	S	S	S	0	0	0	0	0	0	0	0	0	0
10	-	S	S	0	0	0	0	0	0	0	0	0	0
127	S	S	S	0	0	0	0	0	0	0	0	0	0
95	S	S	S	0	0	0	0	0	0	0	0	0	0
1193	S	S	I+R	0	0	0	0	1	0	0	0	1	1
69	S	I+R	S	0	0	0	0	1	1	0	0	1	0
131	S	S	S	0	0	0	0	0	0	0	0	0	0
131	S	I+R	S	0	0	0	1	1	1	1	0	1	0
73	S	S	S	0	0	0	0	1	0	0	0	0	0
7428	S	S	S	0	0	0	0	0	0	0	0	0	0
95	S	S	S	0	0	0	0	1	0	0	0	0	0
73	S	S	S	0	0	0	0	0	0	0	0	0	0
73	S	S	S	0	0	0	0	0	0	0	0	0	0
69	S	S	S	0	0	0	0	0	0	0	0	0	0
12	S	S	S	0	0	1	0	0	0	0	0	1	0
345	S	S	S	0	0	0	0	0	0	0	0	0	0
95	S	S	S	0	0	0	0	0	0	0	0	0	0
73	S	S	S	0	0	0	0	0	0	0	0	0	0
127	S	S	S	0	0	0	0	0	0	0	0	0	0
131	S	S	S	0	0	0	0	0	0	0	0	0	0
1193	S	S	I+R	0	0	0	0	1	0	0	0	1	1

95	S	S	S	0	0	0	0	1	0	0	0	0	0
929	S	S	S	0	0	0	0	0	0	0	0	0	0
73	-	S	S	0	0	0	0	0	0	0	0	0	0
131	S	I+R	I+R	0	0	0	0	1	1	0	0	1	1
69	S	I+R	S	0	0	0	0	1	1	0	0	1	0
1262	S	S	S	0	0	0	0	0	0	0	0	0	0
10	S	S	S	0	0	0	0	0	0	0	0	0	0
73	S	S	S	0	0	0	0	0	0	0	0	0	0
38	S	S	I+R	0	0	0	0	0	0	0	0	1	1
131	S	S	S	0	0	0	0	0	0	0	0	0	0
73	S	S	S	0	0	0	0	1	0	0	0	0	0
1193	S	S	I+R	0	0	0	0	1	0	0	0	1	1
95	S	S	S	0	0	0	0	1	0	0	0	1	0
NEW	-	S	S	0	0	0	0	1	0	0	0	0	0
226	S	S	S	0	0	0	0	1	0	0	0	0	0
1057	S	S	S	0	0	0	0	0	0	0	0	0	0
1193	S	S	I+R	0	0	0	0	0	0	0	0	1	1
1623	S	S	S	0	0	0	0	0	0	0	0	0	0
131	S	S	I+R	0	0	0	0	1	0	0	0	1	1
135	S	S	S	0	0	0	0	0	0	0	0	0	0
46	S	I+R	I+R	0	0	0	0	1	1	0	0	0	0
357	S	S	S	0	0	0	0	0	0	0	0	0	0
131	S	I+R	S	0	0	0	0	1	1	0	0	1	0
12	S	S	S	0	0	0	0	0	0	0	0	0	0
131	S	S	S	0	0	0	0	1	0	0	0	0	0
68	S	S	S	0	0	0	0	0	0	0	0	0	0
95	S	S	S	0	0	0	0	1	0	0	0	1	0
409	S	S	S	0	0	0	0	0	0	0	0	0	0
420	S	S	S	0	0	0	0	0	0	0	0	0	0
46	S	S	S	0	0	0	0	1	0	0	0	0	0
131	I+R	I+R	I+R	0	1	0	0	0	1	0	0	1	1
95	S	S	S	0	0	0	0	0	0	0	0	0	0
4260	S	S	S	0	0	0	0	1	0	0	0	0	0

127	S	S	S	0	1	0	0	0	0	1	0	0	0
131	I+R	S	I+R	0	1	0	1	0	0	1	0	1	1
95	S	S	S	0	0	0	0	1	0	0	0	0	0
2013	S	S	S	0	0	0	0	0	0	0	0	0	0
95	S	S	S	0	0	0	0	1	0	0	0	1	0
5022	S	S	S	0	0	0	0	0	0	0	0	0	0
131	I+R	S	I+R	0	1	0	0	1	0	0	0	1	1
69	S	S	S	0	0	0	0	1	0	0	0	1	0
73	S	S	S	0	0	0	0	0	0	0	0	0	0
69	S	S	S	0	0	0	0	1	0	0	0	0	0
1193	S	S	I+R	0	0	0	0	1	0	0	0	1	1
131	S	I+R	S	0	0	0	0	1	1	0	0	0	0
420	S	S	S	0	0	0	0	1	0	0	0	0	0
131	S	S	S	0	0	0	0	1	0	0	0	0	0
10	S	S	S	0	0	0	0	1	0	0	0	0	0
73	S	S	S	0	0	0	1	0	0	0	0	0	0
62	S	S	S	0	0	0	0	1	0	0	0	0	0
73	S	S	S	0	0	0	0	0	0	0	0	0	0
73	S	S	S	0	0	0	0	0	0	0	0	0	0
393	S	S	I+R	0	0	0	0	1	0	0	0	1	1
131	I+R	S	I+R	0	1	0	0	1	0	0	0	1	1
5022	S	S	S	0	0	0	0	0	0	0	0	0	0
95	S	S	S	0	0	0	0	0	0	0	0	0	0
131	I+R	S	I+R	0	1	0	1	1	0	1	0	1	1
12	I+R	S	S	0	1	0	0	0	0	0	0	1	0
95	S	S	S	0	0	0	0	0	0	0	0	0	0
12	S	S	S	0	0	0	0	0	0	0	0	0	0
68	S	S	S	0	0	0	0	0	0	0	0	0	0
131	I+R	S	I+R	0	1	0	0	0	0	0	0	1	1
131	I+R	S	I+R	0	1	0	1	1	0	1	0	1	1
NEW	S	S	S	0	0	0	0	0	0	0	0	0	1
95	S	S	S	0	0	0	0	0	0	0	0	0	0
131	I+R	I+R	I+R	0	1	0	1	1	1	1	0	1	1

602	S	S	S	0	0	0	0	0	0	0	0	0	0
144	S	S	S	0	0	0	0	1	0	0	0	0	0
648	I+R	I+R	I+R	0	1	0	0	0	1	0	0	1	1
69	S	S	S	0	0	0	0	1	0	0	0	1	0
58	I+R	S	S	1	0	0	0	1	0	0	0	0	0
428	S	S	S	0	0	0	0	0	0	0	0	0	0
59	S	S	S	0	0	0	0	1	0	0	0	0	0
144	S	S	S	0	0	0	0	0	0	0	0	0	0
131	I+R	S	I+R	0	1	0	1	1	0	1	0	1	1
73	S	S	S	0	0	0	0	0	0	0	0	0	0
141	S	S	S	0	0	0	0	0	0	0	0	0	0
73	S	S	S	0	0	0	0	0	0	0	0	0	0
405	I+R	S	I+R	0	1	0	0	0	0	0	0	1	1
38	I+R	S	I+R	0	1	0	0	0	0	0	0	1	1
59	S	S	S	0	0	0	0	0	0	0	0	0	0
69	S	S	S	0	0	0	0	1	0	0	0	0	0
405	I+R	I+R	I+R	0	1	0	0	1	1	0	0	1	1
1161	S	S	S	0	0	0	0	0	0	0	0	0	0
131	S	I+R	S	0	0	0	0	1	1	0	0	1	0
1193	S	S	I+R	0	0	0	0	1	0	0	0	1	1
95	S	S	S	0	0	0	0	0	0	0	0	0	0
73	S	S	S	0	0	0	0	0	0	0	0	0	0
NEW	S	S	S	0	0	0	0	0	0	0	0	0	0
131	S	S	S	0	0	0	0	1	0	0	0	1	0
131	S	S	I+R	0	0	0	0	1	0	0	0	1	1
95	S	S	S	0	0	0	0	1	0	0	0	0	0
73	S	S	S	0	0	1	0	0	0	0	0	0	0
-	I+R	I+R	S	-	-	-	-	-	-	-	-	-	-
491	S	S	S	0	0	0	0	0	0	0	0	0	0
1598	I+R	S	S	0	1	0	0	0	0	0	0	0	0
131	S	S	I+R	0	0	0	0	0	0	0	0	1	1
73	S	S	S	0	0	1	0	0	0	0	0	0	0
73	S	S	S	0	0	1	0	0	0	0	0	0	0

38	S	S	S	0	0	0	0	1	0	0	0	1	0
59	S	S	S	0	0	0	0	0	0	0	0	0	0
399	S	S	S	0	0	0	0	0	0	0	0	0	0
73	S	S	S	0	0	0	0	0	0	0	0	0	0
998	S	S	S	0	0	0	0	1	0	0	0	0	0
131	S	S	S	0	0	0	0	0	0	0	0	0	0
69	S	S	S	0	0	0	0	1	0	0	0	1	0
69	S	S	S	0	0	0	0	1	0	0	0	0	0
349	S	S	S	0	0	0	1	0	0	0	0	1	0
69	S	S	S	0	0	0	0	0	0	0	0	0	0
607	S	S	S	0	0	0	0	0	0	0	0	0	0
38	I+R	S	I+R	0	1	0	0	0	0	0	0	1	1
73	S	S	S	0	0	0	0	0	0	0	0	0	0
393	S	S	I+R	0	0	0	0	0	0	0	0	1	1
2552	S	S	S	0	0	0	0	0	0	0	0	0	0
131	S	S	I+R	0	0	0	0	1	0	0	0	1	1
95	S	S	S	0	0	0	0	0	0	0	0	0	0
93	S	S	S	0	0	0	0	0	0	0	0	0	0
131	S	S	S	0	0	0	0	1	0	0	0	1	0
131	S	I+R	I+R	0	0	0	0	1	1	0	0	1	1
676	S	S	S	0	0	0	0	0	0	0	0	0	0
73	S	S	S	0	0	0	0	0	0	0	0	0	0
131	S	I+R	I+R	0	0	0	0	1	1	0	0	1	1
73	S	S	S	0	0	0	0	0	0	0	0	0	0
131	S	I+R	S	0	0	0	0	1	1	0	0	1	0
14	-	S	S	0	0	0	0	1	0	0	0	1	0
405	I+R	S	I+R	0	1	0	0	0	0	0	0	1	1
95	S	S	S	0	0	0	0	0	0	0	0	0	0
131	S	S	I+R	0	0	0	0	1	0	0	0	1	1
NEW	S	S	S	0	0	0	0	0	0	0	0	0	0
73	S	S	S	0	0	0	0	0	0	0	0	0	0
131	I+R	S	I+R	0	1	0	0	0	0	0	0	1	1
140	S	S	S	0	0	0	0	0	0	0	0	0	0

135	S	S	S	0	0	0	0	0	0	0	0	0	0
95	S	I+R	S	0	0	0	0	0	1	0	0	1	0
62	S	S	S	0	0	0	0	0	0	0	0	0	0
95	S	S	S	0	0	0	0	0	0	0	0	0	0
127	S	I+R	S	0	0	0	0	1	0	0	0	0	0
131	S	S	I+R	0	0	0	0	1	0	0	0	1	1
95	S	S	S	0	0	0	0	1	0	0	0	1	0
919	S	S	S	0	0	0	0	0	0	0	0	0	0
95	S	S	S	0	0	0	0	0	0	0	0	0	0
131	S	S	I+R	0	0	0	0	1	0	0	0	1	1
80	S	S	S	0	0	0	0	0	0	0	0	0	0
617	I+R	S	I+R	0	1	0	0	0	0	0	0	1	1
90	S	I+R	I+R	0	0	0	0	0	1	0	0	1	1
1193	S	I+R	I+R	0	0	0	0	1	1	0	0	1	1
95	S	S	S	0	0	0	0	0	0	0	0	0	0
93	S	S	S	0	0	0	0	0	0	0	0	0	0
297	S	S	S	0	0	0	0	0	0	0	0	0	0
131	S	S	S	0	0	0	0	1	0	0	0	0	0
648	I+R	S	I+R	0	1	0	1	0	0	1	0	1	1
127	S	S	S	0	0	0	0	0	0	0	0	0	0
127	S	S	S	0	0	0	0	0	0	0	0	1	0
73	S	S	S	0	0	0	0	0	0	0	0	0	0
95	S	S	S	0	0	0	0	0	0	0	0	1	0
69	S	S	S	0	0	0	0	0	0	0	0	0	1
95	S	S	S	0	0	0	0	1	0	0	0	0	0
131	I+R	S	I+R	0	1	0	1	0	0	1	0	1	1
12	S	S	S	0	0	1	0	0	0	0	0	0	0
95	S	S	S	0	0	0	0	0	0	0	0	0	0
648	S	I+R	I+R	0	0	0	0	1	1	0	0	1	1
73	S	S	S	0	0	1	0	0	0	0	0	0	0
131	S	S	I+R	0	0	0	0	0	0	0	0	1	1
131	S	S	I+R	0	0	0	0	1	0	0	0	1	1
1177	S	S	S	0	0	0	0	1	0	0	0	1	1

131	S	S	I+R	0	0	0	0	0	0	0	0	1	1
131	S	S	I+R	0	0	0	0	0	0	0	0	1	1
95	S	S	S	0	0	0	0	0	0	0	0	0	0
131	S	S	I+R	0	0	0	0	0	0	0	0	1	1
131	S	S	I+R	0	0	0	0	1	0	0	0	1	1
95	S	S	S	0	0	0	0	0	0	0	0	0	0
95	S	S	S	0	0	0	0	0	0	0	0	0	0
69	S	S	S	0	0	0	0	1	0	0	0	0	0
73	S	S	S	0	0	0	0	0	0	0	0	0	0
62	S	S	S	0	0	0	0	0	0	0	0	0	0
1193	S	S	I+R	0	0	0	0	1	0	0	0	1	1
69	S	S	S	0	0	0	0	1	0	0	0	0	0
1231	S	S	S	0	0	0	0	0	0	0	0	0	0
648	S	S	S	0	0	0	0	0	0	0	0	0	0
93	S	S	S	0	0	0	0	0	0	0	0	0	0
6975	S	S	S	0	0	0	0	0	0	0	0	0	0
538	S	S	S	0	0	0	0	0	0	0	0	0	0
648	I+R	I+R	I+R	0	1	0	1	1	1	1	0	1	1
95	S	S	S	0	0	0	0	0	0	0	0	0	0
-	I+R	I+R	I+R	0	0	1	0	0	0	0	0	-	-
95	S	S	S	0	0	0	0	1	0	0	0	0	0
69	S	S	S	0	0	0	0	1	0	0	0	0	0
73	S	S	S	0	0	0	0	0	0	0	0	0	0
1882	S	S	S	0	0	0	0	0	0	0	0	0	0
349	S	S	S	0	0	0	0	1	0	0	0	0	0
131	S	S	I+R	0	0	0	0	1	0	0	0	1	1
547	S	S	S	0	0	0	0	0	0	0	0	0	0
59	S	S	S	0	0	0	0	0	0	0	0	0	0
NEW	S	S	S	1	0	0	0	0	0	0	0	0	0
537	S	S	S	0	0	0	0	0	0	0	0	0	0
131	S	I+R	I+R	0	0	0	0	1	1	0	0	1	1
95	S	S	S	0	0	0	0	0	0	0	0	0	0
10	S	S	S	0	0	0	0	1	0	0	0	0	0

405	S	S	I+R	0	0	0	0	1	0	0	0	1	1
127	S	S	S	0	0	0	0	0	0	0	0	0	0
131	I+R	I+R	I+R	0	1	0	1	0	1	1	0	1	1
3045	S	S	S	0	0	0	0	0	0	0	0	0	0
73	S	S	S	0	0	0	0	0	0	0	0	0	0
131	S	S	S	0	0	0	0	1	0	0	0	1	0
73	S	S	S	0	0	0	0	0	0	0	0	0	0
1193	S	I+R	I+R	0	0	0	0	1	1	0	0	1	1
421	S	S	S	0	0	0	0	0	0	0	0	0	0
131	S	S	I+R	0	0	0	0	1	0	0	0	1	1
1193	S	S	I+R	0	0	0	0	1	0	0	0	1	1
537	S	S	S	0	0	0	0	0	0	0	0	0	0
95	S	S	S	0	0	0	0	0	0	0	0	0	0
131	S	S	I+R	0	0	0	0	0	0	0	0	1	1
393	S	S	I+R	0	0	0	0	0	0	0	0	1	1
95	S	S	S	0	0	0	0	0	0	0	0	0	0
1844	S	S	S	0	0	0	0	0	0	0	0	0	0
131	S	I+R	I+R	0	0	0	0	1	1	0	0	1	1
127	S	S	S	0	0	0	0	0	0	0	0	0	0
69	S	S	S	0	0	0	0	1	0	0	0	0	0
NEW	S	S	S	0	0	0	0	0	0	0	0	0	0
73	S	S	S	0	0	0	0	1	0	0	0	0	0
73	S	S	S	0	0	0	0	0	0	0	0	0	0
NEW	I+R	S	S	0	1	0	0	0	0	0	0	0	0
491	S	S	S	0	0	0	0	0	0	0	0	0	0
95	S	S	I+R	0	0	0	0	1	1	0	0	1	1
73	S	S	S	0	0	0	0	1	0	0	0	0	0
69	S	S	S	0	0	0	0	1	0	0	0	0	0
73	S	S	S	0	0	0	0	0	0	0	0	0	0
73	S	S	S	0	0	0	0	1	0	0	0	0	0
95	S	S	S	0	0	0	0	0	0	0	0	0	0
10	S	S	I+R	0	0	0	0	1	0	0	0	1	1
95	S	S	S	0	0	0	0	1	0	0	0	0	0

127	S	S	S	0	0	0	0	1	0	0	0	0	0
428	S	S	S	0	0	0	0	1	0	0	0	0	0
73	S	S	S	0	0	0	0	0	0	0	0	0	0
5969	S	S	S	0	0	0	0	0	0	0	0	0	0
131	-	S	I+R	1	0	0	0	1	0	0	0	1	1
131	S	I+R	I+R	0	0	0	0	1	1	0	0	1	1
69	S	S	S	0	0	0	0	1	0	0	0	0	0
73	S	S	S	0	0	0	0	1	0	0	0	0	0
1236	S	S	S	0	0	0	0	0	0	0	0	0	0
69	S	S	S	0	0	0	0	1	0	0	0	0	0
131	S	S	I+R	0	0	0	0	1	0	0	0	1	1
131	S	S	S	0	0	0	0	1	0	0	0	1	1
131	I+R	I+R	I+R	0	1	0	0	1	1	0	0	1	1
91	S	S	S	0	0	0	0	0	0	0	0	0	0
131	I+R	I+R	I+R	0	1	0	1	0	1	1	0	1	1
95	S	S	S	0	0	0	0	1	0	0	0	0	0
62	S	S	S	0	0	0	0	1	0	0	0	0	0
73	S	S	S	0	0	0	0	0	0	0	0	0	0
-	I+R	S	I+R	0	1	0	0	1	0	0	0	1	0
491	S	S	S	0	0	0	0	0	0	0	0	0	0
1385	S	S	S	0	0	0	0	0	0	0	0	0	0
95	S	S	S	0	0	0	0	0	0	0	0	0	0
69	S	I+R	S	0	0	0	0	1	1	0	0	1	0
131	S	I+R	S	0	0	0	0	1	1	0	0	1	1
73	I+R	S	S	0	0	0	0	0	0	0	0	0	0
95	S	S	S	0	0	0	0	1	0	0	0	0	0
95	S	S	S	0	0	0	0	0	0	0	0	1	0
131	S	I+R	I+R	0	0	0	0	1	1	0	0	1	1
93	S	S	I+R	0	0	0	0	0	0	0	0	1	1
69	S	S	S	0	0	0	0	0	0	0	0	1	0
38	-	I+R	I+R	0	1	0	1	1	1	1	0	1	1
NEW	S	S	S	0	0	0	1	0	0	0	0	0	0
73	S	S	S	0	0	0	0	0	0	0	0	0	0

131	S	S	I+R	0	0	0	0	1	0	0	0	1	1
131	S	S	S	0	0	0	0	1	0	0	0	0	0
127	S	S	S	0	0	0	0	0	0	0	0	0	0
131	S	I+R	I+R	0	0	0	0	1	1	0	0	1	1
131	S	S	S	0	0	0	0	1	0	0	0	0	0
410	I+R	S	I+R	1	0	0	1	1	0	0	0	1	1
95	I+R	S	S	1	0	0	0	0	0	0	0	0	0
95	S	S	S	0	0	0	0	1	0	0	0	0	0
416	-	S	S	0	0	0	0	0	0	0	0	0	0
95	S	S	S	0	0	0	0	0	0	0	0	0	0
1415	S	S	S	0	0	0	0	0	0	0	0	0	0
393	S	I+R	I+R	0	0	0	0	1	1	0	0	1	1
95	S	S	S	0	0	0	0	0	0	0	0	0	0
3672	S	S	S	0	0	0	0	0	0	0	0	0	0
127	S	S	S	0	0	0	0	0	0	0	0	0	0
131	I+R	S	I+R	0	1	0	0	0	0	0	0	1	1
998	I+R	S	S	0	1	0	1	1	0	1	1	1	0
405	I+R	I+R	I+R	1	0	0	0	1	0	0	1	1	1
69	S	S	S	0	0	0	0	1	0	0	0	0	0
127	-	S	S	0	0	0	0	0	0	0	0	0	0
131	S	I+R	I+R	0	0	0	0	1	1	0	0	1	1
405	I+R	I+R	I+R	1	0	0	0	1	0	0	1	1	1
131	I+R	S	I+R	0	1	0	1	0	0	1	0	1	1
95	S	S	S	0	0	0	0	0	0	0	0	1	0
405	I+R	I+R	I+R	1	0	0	0	1	0	0	1	1	1
131	S	S	S	0	0	0	0	1	0	0	0	1	0
73	S	S	S	0	0	0	0	0	0	0	0	0	0
127	S	S	S	0	0	0	0	0	0	0	0	0	0
131	S	I+R	S	0	0	0	0	1	1	0	0	1	0
93	I+R	I+R	S	1	0	0	0	0	1	0	0	0	0
59	-	S	S	0	0	0	0	0	0	0	0	0	0
131	S	I+R	S	0	0	0	0	1	1	0	0	1	0
38	I+R	S	I+R	0	1	0	0	0	0	0	0	1	1

10	S	S	S	0	0	0	0	0	0	0	0	0	0
95	S	S	S	0	0	0	0	0	0	0	0	0	0
131	I+R	S	S	1	0	0	0	1	0	0	0	0	0
405	S	I+R	I+R	0	0	0	1	0	1	1	0	1	1
73	S	S	S	0	0	0	0	0	0	0	0	0	0
131	S	S	I+R	0	0	0	0	0	0	0	0	1	1
349	S	S	S	0	0	0	0	0	0	0	0	0	0
12	S	S	S	0	0	0	0	0	0	0	0	0	0
131	-	S	I+R	0	0	0	0	1	0	0	0	1	1
1193	S	S	I+R	0	0	0	0	1	0	0	0	1	1
95	I+R	S	S	1	0	0	0	0	0	0	0	0	0
131	S	S	S	0	0	0	0	1	0	0	0	1	0
1284	I+R	S	I+R	0	1	0	1	0	0	1	0	1	1
144	S	S	S	0	0	0	0	0	0	0	0	0	0
648	I+R	I+R	I+R	0	1	0	0	1	1	0	0	1	1
95	S	S	S	0	0	0	0	0	0	0	0	0	0
73	S	S	S	0	0	0	0	0	0	0	0	0	0
131	S	I+R	I+R	0	0	0	0	1	1	0	0	1	1
404	S	S	S	0	0	0	0	1	0	0	0	1	0

Supplementary Figure 1: Receiver operating characteristic curves (ROC) for complete antibiotic resistance loci models with and without epidemiologic predictors for (a) 3rd generation cephalosporins (ceftriaxone), (b) fluoroquinolones (ciprofloxacin), and (c) aminoglycosides (gentamicin).

(a)

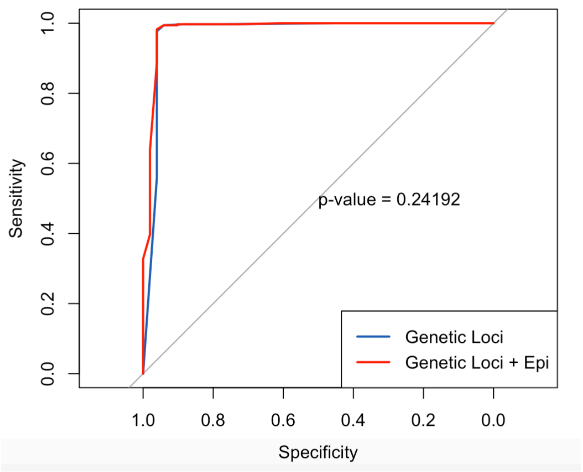

(b)

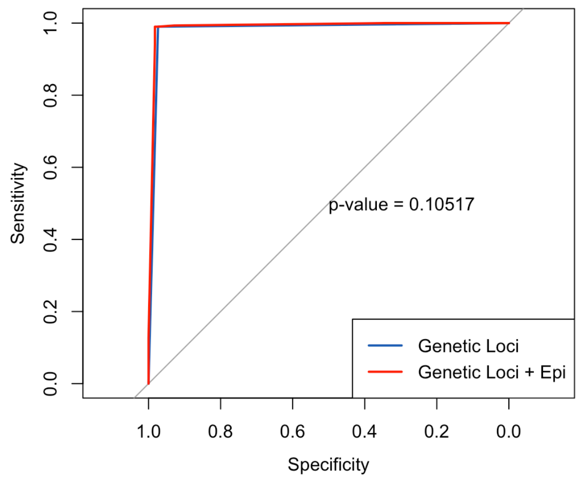

(c)

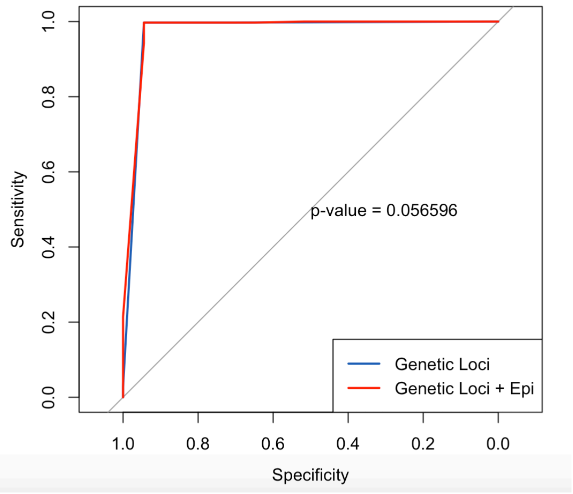

Sensitivity
1.0
0.8
0.6
0.4
0.2
0.0
p-value = 0.056596
Genetic Loci
Genetic Loci + Epi
1.0
0.8
0.6
0.4
0.2
0.0
Specificity
